# Assessment of Knowledge, Attitudes, and Practices of Traditional Healers toward Dosage Forms and Routes of Administration: A Cross-Sectional Study in Ethiopia

**DOI:** 10.1155/2023/7091233

**Published:** 2023-06-27

**Authors:** Liknaw Workie Limenh, Derso Teju Geremew, Asmamaw Emagn Kasahun, Yeniewa Kerie Anagaw, Minichil Chanie Worku, Wudneh Simegn, Wondim Ayenew

**Affiliations:** ^1^Department of Pharmaceutics, School of Pharmacy, College of Medicine and Health Sciences, University of Gondar, Gondar, Ethiopia; ^2^Department of Pharmaceutical Chemistry, School of Pharmacy, College of Medicine and Health Sciences, University of Gondar, Gondar, Ethiopia; ^3^Department of Social and Administrative Pharmacy, School of Pharmacy, College of Medicine and Health Sciences, University of Gondar, Gondar, Ethiopia

## Abstract

**Methods:**

A cross-sectional study design was conducted on 70 traditional healers from June 1, 2022 to July 25, 2022. The data were collected through structured questionnaires. The data were checked for completeness and consistency and then entered into SPSS version 25.0 for analysis. The results were presented with frequencies and percentages. The association between sociodemographic factors and traditional healers' knowledge of dosage forms and route of administration was determined using the Pearson's chi-squares test. A statistically significant difference was declared if the *p* value was 0.05 or lower.

**Results:**

Most (58.1%) traditional healers had information on dosage forms, especially about solid, semisolid, and liquid dosage forms. In addition, 33 (53.2%) traditional healers had information about rectal, nasal, and oral route of administration. All traditional healers had practiced different dosage forms and route of administration both individually and in combination earlier to date. More than half of the participants agreed on the need for different dosage forms and route of administration. This study result also showed that most (72.6%) traditional healers had gaps in sharing experiences and information with other healers and health professionals.

**Conclusions:**

The current study revealed that solid, semisolid, and liquid were the most frequently formulated dosage forms with oral, rectal, and nasal route of administration by traditional healers. The practice of checking the status of the formulations was poor. Traditional healers had a good attitude towards the need for different dosage forms and route of administration. The stakeholders should provide continuous training and exchange of experiences between traditional healers and healthcare professionals to improve the knowledge of traditional healers for appropriate use of dosage forms and route of administration.

## 1. Introduction

Traditional medicine (TM) is a body of knowledge, skills, and practices used to prevent, diagnose, and treat disease, which is developed by indigenous communities [1, 2]. Because TMs are widely accepted, available, accessible, and affordable, many individuals wish they could treat their diseases [3–5]. As reported by the World Health Organization (WHO), 80% of the populations in emerging markets rely on TMs for therapy. It has been argued that the widespread use of TMs in Africa is linked to cultural and economic reasons. For this reason, the WHO encourages African countries to promote and integrate traditional treatment practices into their health systems [2]. In Ethiopia, more than 70% of people rely on TMs for their healthcare [6].

Traditional medicine-dosage forms (TM-DFs) have recently become popular, and for any medical system, both the medications and their dosage forms (DFs) are essential to providing appropriate care [7]. The United States Pharmacopeia (USP) defined DFs as a combination of drug substances and excipients to facilitate the dosing and administration of the drug to the patient in different routes of administration (RoA). The design and testing of all DFs are aimed at drug product quality [8, 9]. These DFs are suspensions, capsules, tablets, creams, ointments, transdermal patches, and so on [10]. The RoA is a means used to introduce medicine, liquid, or other things into the body, which includes parenteral such as intravascular, intramuscular, subcutaneous, and inhalation administration; enteral such as oral, sublingual, and rectal administration; and topically such as skin and mucous membranes [11]. Improved solubility, stability, compliance, bioavailability, pharmacological activity, sustained drug delivery, improved patient outcome, and reduced toxicity should be considered in RoA selection [12, 13]. Herbs and plants can be processed and used in different ways and in different forms such as syrups, ointments, capsules, tablets, and herbal creams [7]. Traditional Chinese use various DFs such as lotions, pastes, powders, and topical sustained-release films [14]. In the Ayurvedic healing system, many DFs are available to satisfy elixir potency to ensure convenience for RoA, palatability, and stability [15]. The RoA of herbal DFs includes oral, rectal, topical, parenteral, nasal, ophthalmic, inhalation, and otic [16, 17].

Attitudes toward DFs are influenced by several factors such as age, experience with particular DFs, health status, and frequency of medication use. In addition, taste and swallowability emerged as important attributes for the choice of medicines. Overall, liquid was the most commonly used DF, especially for children under 12 years of age. Monolithic solid DFs, i.e., tablets and capsules, are mainly chosen by adolescents and children of all ages with chronic health problems [18, 19]. Several factors specific to the pediatric population such as the ability to swallow and palatability issues can hamper the delivery of oral medications [20]. By altering DFs in accordance with the actual requirement, THs may provide TMs to the ailing humanity more effectively [13]. Dimensions and palatability are often modified by cutting the tablets in half to improve swallowability or masking the taste to improve palatability, especially for the elderly and children [18]. The prevalence of parental TM practice in children was high, amounting to 71% in children older than 12 months in Fagita Lekoma Woreda, Northwest Ethiopia [21].

The WHO TM Strategy 2014–2023 promoted the safe and effective use of TMs through the regulation of products, practices, and practitioners. These strategies will be achieved by building the knowledge base and developing national strategies, strengthening safety, quality, and effectiveness through regulation, and promoting universal health coverage through the integration of TM services and self-care into national health systems [1]. Therefore, in order to ensure the TM strategy, THs should have adequate knowledge of the formulations and routes associated with the effect on their patients and their treatment outcomes. Inappropriate use of TM, DFs, and RoA leads to toxicity, ineffectiveness, noncompliance, and generally poor patient outcomes. This study aimed to assess the knowledge, attitudes, and practice of various DFs and RoA by THs.

## 2. Materials and Methods

### 2.1. Study Design Period

A cross-sectional study design was conducted from June 1, 2022 to July 25, 2022.

### 2.2. Study Area

The study was conducted in the Amhara region, Northwest Ethiopia. Ethiopia has 9 regions in total, with the Amhara region being one of them. Its population makes up about one-third of all Ethiopians. The Amhara region has an estimated area of 154,708.96 km^2^ and is bordered by Sudan to the west and northwest. It also bordered other regions of Ethiopia: Oromia to the south, Tigray to the north, Afar to the east, Benishangul-Gumuz to the west, and southwest. Based on the 2007 census conducted by the Central Statistical Agency of Ethiopia, the Amhara region has a population of 17,221,976 with a GR of 1.7, of which 8,641,580 are males and 8,580,396 are females [22]. According to the Ethiopian Food and Drug Administration (EFDA), 422 registered THs have been found in the Amhara region. The Amhara region was selected as a study area because of the known history of having traditional medicine practices, and it may be favorable for us to increase representation.

### 2.3. Source and Study Population

All THs in the Amhara region were the source population. The study population consisted of THs who were registered and practiced their work for more than one year. THs that were not voluntary to participate and refused to provide informed consent were excluded from the study.

### 2.4. Sample Size and Sampling Techniques

We recognized TH's workplace as a healthcare facility. Fifteen percent of the THs facilities found in the Amhara region were included because the Logistic Indicator Assessment Tool (LIAT) (USAID|DELIVER) advised a minimum of 15% of healthcare facilities be considered to evaluate the facilities in a specific area when resource restriction is a concern.

(1) $$\begin{array}{c} n=N*0.15\text{,} \end{array}$$

where *n* = the required sample size. *N* = total number of THs facilities (traditional healers) in the region which were 422. *n* = 422 *∗* 0.15 = 63.3, when we use a nonresponse rate of 10%, the sample size equals 69.9. The final sample size included 70 TH facilities. The participants were selected using systematic random sampling. The authors calculated the *K*^th^ value (422/70), and the first study participant was selected by lottery method. At each of the 70 TH facilities, the main TH professional was employed.

### 2.5. Study Variables

The dependent variables of the study were the knowledge, attitude, and practice of THs toward DFs and RoA. Sociodemographic characteristics of the participants such as gender, age, religion, marital status, level of education, and years of experience were used as independent variables.

### 2.6. Data Collection Instrument and Procedure

Data collection was carried out using interview-based questionnaires, which were developed based on reviewing different literature. The questionnaires contained a list of common DFs and RoA, their status from the past to the present, and ways of learning about DFs and RoA. Moreover, the questionnaires also contained questions that focused on healers' attitudes, and practices regarding DFs and RoA during traditional treatments [23–26]. The five-point Likert scale (strongly disagree, disagree, neutral, agree, and strongly agree) was used to assess attitudes toward DFs and RoA, and a three-point Likert scale (always, sometimes, and never) was used to assess the practice of THs towards DFs and RoA. A total of three pharmacists were assigned for data collection. To reduce any potential research bias during the study, data were collected in person at the working area of the THs.

### 2.7. Data Quality Control

The questionnaire was first prepared in English and translated into Amharic and then back into English to check the originality of the message. The data collectors received a half-day training session to explain the purpose and relevance of the study. Before starting the actual data collection, a pretest was performed on 10 THs out of the study are (Afar region) in a similar setup to ensure the consistency and clarity of the data collection tool. Then, tools and procedures were revised based on the pretest performed and data were collected with daily checks for completeness and quality.

### 2.8. Data Processing and Analysis

The data were entered into SPSS software version 25.0 for analysis. Tables and figure were used to present the descriptive part of the findings with frequencies and percentages. The association between sociodemographic factors and THs' knowledge of DFs and RoA were determined using the Pearson's chi-squares test. A statistically significant difference was declared if the *p* value was 0.05 or lower.

## 3. Results

### 3.1. Sociodemographic Characteristics of THs

In the current study, 62 participants were included with a response rate of 88.57%. Of all participants, 60 (96.8%) were males and two (3.2%) were females. The mean age of the participants was 46.23 years with a standard deviation of 0.12. Of the participants, about 31 (50%) had no formal education (illiterate) and only 27.4% had completed elementary and secondary education. Regarding religion, 52 (83.9%) of the participants were followers of Orthodox Christianity, and only 10 (16.1%) were Muslims. Regarding the marital status of the participants, 51 (82.3%) were married, four (6.5%) were single, four (6.5%) were widowed, and three (4.8%) were divorced. Their work experience in TM practice ranged from 1 to 41 years with a mean of 21.05 years and a standard deviation of 0.06 (Table 1).

### 3.2. Traditional Healers' Knowledge and Usage of DFs and RoA

Regarding THs information about DFs, more than half (58.1%) of them had a high awareness on solid, semisolid, and liquid DFs. About 12.9% of THs also knew gaseous along with the previously listed DFs. About two (3.2%) THs of each group only had information on liquid and solid DFs. In terms of RoA, more than half (53.2%) of THs had information about rectal, nasal, and oral RoA. About seven (11.3%) of the THs also had information on parenteral, rectal, nasal, and oral RoA. Only two (3.2%) THs only had information on single RoA such as nasal and oral RoA (Table 2).

All THs had previously practiced various DFs and RoAs both individually and in combination. About 36 (58.1%) of the THs used solid, semisolid, or liquid DFs most frequently in the past. About eight (12.9%) of THs have also previously used solids, semisolids, liquid, and gaseous DFs. Solid and liquid DFs were the only forms previously used individually by THs, although only 3.2%. Rectal, nasal, and oral routes are the most common (53.2%) ways to deliver drugs to patients with THs. Some (3.2%) THs used single RoA types such as nasal and oral (Table 3).

The majority of THs (36 (58.1%)) currently use solid, semisolid, and liquid DFs in combination. Some THs employed only one form of DF such as two (3.2%) of the THs used solid and four (6.4%) of the THs used liquid. More than half (53.2%) of THs current use of RoA was mainly dominated by rectal, nasal, and oral routes. Oral and nasal RoA were the only routes used individually by some (3.2%) THs (Table 4).

Using the Pearson's chi-squares test and a *p* value of 0.05, it was determined that most sociodemographic characteristics had no significant impact on THs' knowledge of DFs and RoA. However, the experience was significantly (*p*=0.016) associated with TH awareness of DFs and RoA. Their knowledge also improved as their experiences increased (Table 1).

### 3.3. Traditional Healers' Sources of Knowledge about Traditional Treatment, DFs, and RoA

More than half of THs (51.6%) obtained their knowledge of the use of TMs, DFs, and RoA from their family (it was in the selective form of generation-to-generation transmission). Seven (11.3%) of them acquired their knowledge of traditional practices from their religious school, and five (8.1%) of them acquired and deepened their knowledge through the combined use of pharmacy counseling, television, family, and religious school. Only two (3.2%) of the THs acquired their knowledge through modern education. Only 27 (43.5%) of study participants were received training on DFs and RoA from the stakeholder capacity building of THs (Table 5).

### 3.4. Traditional Healers' Attitude toward DFs and RoA

In this study, over half (69.4%) of the participants agreed that different DFs and RoA are necessary for different cases. More than half (53.2%) of the study participants agreed that the best method to treat different diseases was the use and application of different kinds of DFs and RoA. About 59.7% of the study participants agreed that appropriate DFs and RoA can offer the patient appropriate medication. In addition, the majority (58.1%) of the participants strongly disagreed with DFs and RoA only required in modern health facilities, which was similar to the above agreement results. About 41.9% of participants had a neutral attitude to TH and should prepare and use different kinds of DFs and RoA. In general, more than half (65.9%) of the participants had favorable attitudes toward different DFs and RoA (Table 6).

### 3.5. Traditional Healers' Practices regarding DFs and RoA

**Figure 1:**
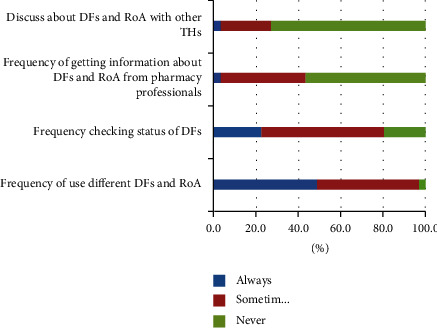
Traditional healers' practices towards dosage forms and routes of administration (*n* = 62).

Thirty (48.4%) of the THs occasionally used various DFs and RoA, which corresponded to the regular users. The remaining (3.2%) THs gave their raw materials to their patients and the patients made the formulations themselves. Regarding the TH checkup status of DFs, only 22.6% THs follow their formulations regularly. About 36 (58.1%) of them inspected the status of their drug preparations intermittently, and 12 (19.4%) of them never checked their formulations unless they desired to dispense them. This study also found that most THs had large gaps in sharing experiences and information with other healers and health professionals. About 56.5% of THs did not communicate with medical and pharmaceutical professionals, and 72.6% of them did not communicate and were unwilling to communicate with other healers (Figure 1).

## 4. Discussion

This study attempted to assess the knowledge, attitudes, and practices of THs toward different DFs and RoA in the Amhara region. Nearly all of our responders (96.8%) were males, which were comparable to the study conducted in Cameron (100% males) [27]. This might be associated with the parent preference of males for teaching traditional treatment practices since THs major source of knowledge was family. In terms of age, our study participants were elderly, comparable to a THs study conducted in Iran, and a large number of them were older than 50 years [28]. Based on educational status, most of our participants were illiterate, which was inconsistent with Cameroonian THs (majority of them had primary education (83.33%)) [29], and THs from Ghana 86.75% of THs had completed elementary and higher education [30]. Most of our participants were either illiterate or gained traditional education that might also be related to their practices and sources of knowledge. The experience of THs was comparable to that of THs in the city of Gondar in north-western Ethiopia, where the majority of healers (78.6%) had an experience of more than 10 years. Moreover, the religious features of Gondar were similar to our results, which showed that a large proportion of THs practiced Ethiopian Orthodox Christianity (92.9%) and only (7.1%) represented Muslims [31].

Our participants prepared different types of formulations and used different RoAs. Their information about different DFs and RoA, their usage history of different types of DFs and RoA, and their current usage of DFs and RoA were linked together. These showed that awareness of different formulations and RoA increases the use status of DFs and RoA. Most of them commonly developed and used solid, semisolid, and liquid DFs with rectal, nasal, and oral administration comparable to traditional Persian pharmacy. The last list of common DFs include solid, semisolid, liquid, and gaseous forms with oral, topical administration, nasal, parenteral, vaginal, ophthalmic, and rectal RoA [32–34]. Tabuti et al. noted that the oral route was commonly practiced by THs [34]. Although there were additional specialized DFs such as vaginal pessaries, rectal, and some elementary inhalants in Iranian THs, our participants also used different topical and oral DFs like Iranian THs [28, 35, 36]. THs in Sri Lanka have used many external applications to treat wounds with different DFs, such as liquid, solid, and gaseous wounds, which are similar formulation types to our participants [37].

When we compared our participants with THs found in different parts of Ethiopia, we also obtained comparable results. These results illustrated that many preparation and administration statuses were nearly similar. According to a study conducted at Gondar THs, oral, topical, and inhalation RoA were often administered by them using solid and liquid formulation techniques. They also formulated drugs in the form of pastes for topical RoA [30]. Liquid DFs were also commonly applied locally and internally [38]. Moreover, an investigation conducted by Gedif and Hahn showed 54.9% of DFs were liquid, followed by unprocessed herbs (12.9%) with varied RoA according to the DFs and their intended purpose [39].

Most of our participants learned their traditional treatment practices from their families, although they used further work such as reading and gaining advice from stakeholders to expand their practices. This result was similar to most countries' THs. For instance, Indonesian THs have learned their TM customs from their parents, family, and books [40]. Moreover, in Limpopo Province, South Africa, 50% of THs acquired their traditional healing knowledge from their parents, and 34% THs became healers through the mentoring of another healer [41].

Next to family, the second-most source of knowledge for our participants was religious schools and books, either in conjunction with previous sources or as individual sources. Our findings were similar to those studied at Gondar city THs. Their most origin of knowledge was from family (57.1%), followed by religious institutions (21.4%) and friends (14.3%). Pharmacy advice, friends, radio, television, and newspapers were additional sources of knowledge used by our participants to expand their traditional treatment practices. Regarding their experience and information sharing about their treatment, our participants had a poor habit of communicating. The result was comparable to Gondar City THs, which showed that most healers kept their knowledge of healing practices secret [30]. This habit is also worsened by their paucity of gaining communication with pharmacy professionals or other health professionals. However, these results were in contrast with a study done by Gedi and Hahn, which revealed that the majority of the examined healers expressed their general readiness to work in close collaboration with modern medicine practitioners, although most healers were observed to have no interplay with modern practitioners [39].

Our study participants' practice in terms of formulation review and knowledge of instability issues was poor, perhaps due to their paucity of knowledge about instability the problems of various DFs and associated side effects. Our finding was in contrast to THs in Limpopo Province, South Africa, where 80% of THs reported prepared remedies deteriorating, which is a temperature-dependent process. They checked and knew what conditions made their products unstable, and they controlled those factors. 75% of THs in South Africa reported that liquid medicines stored in a hot environment expire within a week or even less, while those stored in cooler locations are kept and checked continuously. They also knew the attributes of expired liquid medicines such as color change, coagulation of liquid, DFs, and odor generation. They also knew that solid medicines have a longer shelf life [41].

## 5. Limitations of the Study

While this investigation is of the utmost importance to indicate future work on this subject by researchers and all stakeholders, it has limitations. Such limitations are a small size and inadequate availability of comparable data. The inadequate availability of data, especially attitude and practice-related data toward DFs and RoA, complicated our comparison and discussion. The study recognized that the information provided by THs by owners of traditional treatment facilities may not be accurate and introduced some subjective bias, although observational techniques were used to reduce bias. Furthermore, because of privacy concerns, the participants may not have provided enough information to evaluate the type of medication; therefore, we solely focused on DFs and RoA.

## 6. Conclusions

There are different types of DFs and RoA, each with unique advantages and disadvantages that should be considered when treating patients in both modern and conventional healthcare systems. For oral, rectal, and nasal RoA, our participants most commonly formulated and used solid, semisolid, and liquid DFs. They will use DFs and RoA appropriately as they improve their expertise and collaborate with other THs and modern healthcare professionals, which helps reduce the negative impact and ineffectiveness caused by improper use of DFs and RoA. In addition, expanding continuing education and sharing experiences between THs and healthcare professionals would improve the standards and security of DFs and the applicability of RoA. Given that conventional treatments are of tremendous benefit worldwide, the importance of improving the status of the use of DFs and RoA should be emphasized by all stakeholders. DFs and RoA have been ignored as study areas both domestically and internationally. There is a need for scientific studies to identify gaps in the use of DFs and RoA, educate THs on the proper use of DFs and RoA, and inform them of any risks that could result from improper use of DFs and RoA. Upon completion of this study, commonly used DFs and RoA were identified. By focusing on this, THs will receive training on the pros and cons of the various DFs and RoA they use, as well as the circumstances and methods by which they choose the formulations and route types they use.

## Figures and Tables

**Table 1:** Sociodemographic characteristics of traditional healers (*n* = 62).

Variables	Categories	Frequency (%) (*N* = 62)	Heard about different types of DFs and RoA N (%)	Not heard about different types of DFs and RoA N (%)	Statistical tests
Gender	Male	60 (85.7)	52 (86.7)	8 (13.3)	*X* ^2^ = 0.306 *p*=0.580
	Female	2 (2.86)	2 (100.0)	0 (0.0)	

Age	Under 30	4 (5.71)	2 (50.0)	2 (50.0)	*X* ^2^ = 5.518 *p*=0.138
	31–40	14 (20.0)	12 (85.7)	2 (14.3)	
	41–50	20 (28.6)	18 (90.0)	2 (10.0)	
	Over 50	24 (34.3)	22 (91.7)	2 (8.3)	

Education level	Illiterate	31 (44.3)	25 (80.6)	6 (19.4)	*X* ^2^ = 3.690 *p*=0.297
	Primary	8 (11.4)	8 (100.0)	0 (0.0)	
	Secondary	9 (12.9)	9 (100.0)	0 (0.0)	
	Tertiary	14 (20.0)	12 (85.7)	2 (14.3)	

Religion	Orthodox	52 (74.3)	46 (88.5)	6 (11.5)	*X* ^2^ = 0.534 *p*=0.465
	Muslim	10 (14.3)	8 (80.0)	2 (20.0)	

Marital status	Married	51 (72.9)	45 (88.2)	6 (11.8)	*X* ^2^ = 5.994 *p*=0.112
	Divorced	3 (4.29)	3 (100.0)	0 (0.0)	
	Single	4 (5.71)	2 (50.0)	2 (50)	
	Widowed	4 (5.71)	4 (100.0)	0 (0.0)	

Experience (in years)	Under 5	2 (2.86)	2 (100.0)	0 (0.0)	*X* ^2^ = 8.282 *p*=0.016
	6–10	6 (8.57)	3 (50.0)	3 (50)	
	Over 10	54 (77.1)	49 (90.7)	5 (9.3)	

**Table 2:** Traditional healers' knowledge about dosage forms and routes of administration (*n* = 62).

Questions	Responses	Frequency	Percentage (%)
Which type of DFs have you heard of	Liquid	4	6.4
	Solid, semisolid, liquid, and gaseous	8	12.9
	Solid, semisolid, and liquid	36	58.1
	Solid and semisolid	6	9.7
	Solid	2	3.2
	Semisolid and liquid	6	9.7

Which type of RoA have you heard of	Nasal	2	3.2
	Inhalational, rectal, nasal, and oral	6	9.7
	Rectal, nasal, and oral	33	53.2
	Parenteral, rectal, nasal, and oral	6	9.7
	Parenteral, rectal, and oral	4	6.5
	Rectal and oral	2	3.2
	Nasal and oral	7	11.3
	Oral	2	3.2

**Table 3:** History of using dosage forms and routes of administration by traditional healers (*n* = 62).

Questions	Responses	Frequency	Percentage (%)
Type of DFs used before	Solid, semisolid, liquid, and gaseous	8	12.9
	Solid, semisolid, and liquid	36	58.1
	Solid and semisolid	6	9.7
	Semisolid and liquid	6	9.7
	Liquid	4	6.4
	Solid	2	3.2

Type of RoA used before	Nasal	2	3.2
	Inhalational, rectal, and nasal, oral	6	9.7
	Rectal, nasal, and oral	33	53.2
	Parenteral, rectal, nasal, and oral	6	9.7
	Parenteral, rectal, and oral	4	6.5
	Rectal and oral	2	3.2
	Nasal and oral	7	11.3
	Oral	2	3.2

**Table 4:** Current usage status of dosage forms and routes of administration by traditional healers (*n* = 62).

Questions	Responses	Frequency	Percentage (%)
Types of current DFs used	Solid, semisolid, liquid, and gaseous	8	12.9
	Solid, semisolid, and liquid	36	58.1
	Solid and semisolid	6	9.7
	Semisolid and liquid	6	9.7
	Liquid	4	6.4
	Solid	2	3.2

Type current RoA used	Nasal	2	3.2
	Inhalational, rectal, nasal, and oral	6	9.7
	Rectal, nasal, and oral	33	53.2
	Parenteral, rectal, nasal, and oral	8	12.9
	Parenteral, rectal, and oral	2	3.2
	Rectal and oral	2	3.2
	Nasal and oral	7	11.3
	Oral	2	3.2

**Table 5:** Traditional healers' way of learning about traditional treatment, dosage forms, and routes of administration (*n* = 62).

Questions	Response	Frequency	Percentage (%)
Way of learning traditional treatment practices, DFs and RoA	Pharmacy advice	4	6.5
	Family	32	51.6
	Religious school	7	11.3
	Pharmacy advice, friend, radio, television, newspapers, family, and religious school	2	3.2
	Pharmacy advice, television, family, and religious school	5	8.1
	Friend and family	4	6.5
	Pharmacy advice and family	2	3.2
	Friend, radio, television, family, and religious school	2	3.2
	Modern education	2	3.2
	Pharmacy advice, friend, and radio	2	3.2

Training about DFs and RoA	Yes	27	43.5
	No	35	56.5

**Table 6:** Attitude of traditional healers towards dosage forms and routes of administration (*n* = 62).

Attitude questions	Responses (*n* (%))				
	Strongly disagree	Disagree	Neutral	Agree	Strongly agree
Different DFs and RoA are necessary for different cases	2 (3.2%)	0 (0.0%)	0 (0.0%)	43 (69.4%)	17 (27.4%)
Best way to treat different disease is use of different types of DFs and RoA	2 (3.2%)	4 (6.5%)	6 (9.7%)	33 (53.2%)	17 (27.4%)
Using appropriate DFs and RoA can provide my patient appropriate medication	2 (3.2%)	0 (0.0%)	0 (0.0%)	37 (59.7%)	23 (37.1%)
I am sure that I can use appropriately any DFs and RoA	8 (12.9%)	7 (11.3%)	14 (22.6%)	13 (21.0%)	20 (32.3%)
Only some medicines needs different DFs and RoA	2 (3.2%)	12 (19.4%)	16 (25.8%)	24 (38.7%)	8 (12.9%)
It is dangerous in medication use when different DFs and RoA are not used	8 (12.9%)	9 (14.5%)	14 (22.6%)	19 (30.6%)	12 (19.4%)
I can prepare and use any type of DFs and RoA	13 (21.0%)	10 (16.1%)	16 (25.8%)	11 (17.7%)	12 (19.4%)
I will seek for advice if I want to use new DFs and RoA	24 (38.7%)	9 (14.5%)	2 (3.2%)	19 (30.6%)	8 (12.9%)
All TH should prepare and use different types of DFs and RoA	4 (6.5%)	4 (6.5%)	26 (41.9%)	23 (37.1%)	5 (8.1%)
Using various DFs and RoA requires a special knowledge	9 (14.5%)	21 (33.9%)	12 (19.4%)	4 (6.5%)	16 (25.8%)
DFs and RoA only necessary in modern health facilities	36 (58.1%)	22 (35.5%)	2 (3.2%)	2 (3.2%)	0 (0.0%)
I am well informed about DFs and RoA	4 (6.5%)	9 (14.5%)	16 (25.8%)	15 (24.2%)	18 (29.0%)

## Data Availability

All data generated during this study are included in this published article. All materials and data related to our study are not available publicly, but they will be available from the publisher if the corresponding author is asked to share them.

## Abbreviations

CAM: Complementary or alternative medicine
DF: Dosage forms
EFDA: Ethiopian Food and Drug Authority
RoA: Routes of administration
TH: Traditional healers
TM: Traditional medicine
USP: United States Pharmacopeia
WHO: World Health Organization.
